# Analysis of the action mechanisms and targets of herbal anticonvulsants highlights opportunities for therapeutic engagement with refractory epilepsy

**DOI:** 10.1007/s00109-024-02445-5

**Published:** 2024-04-24

**Authors:** Sobia Tabassum, Susan Shorter, Saak V. Ovsepian

**Affiliations:** 1 Department of Biological Sciences, Faculty of Sciences, International Islamic University, Islamabad, Pakistan; 2 Faculty of Engineering and Science, University of Greenwich London, Chatham Maritime, Kent, ME4 4TB UK; 3 Faculty of Medicine, Tbilisi State University, Tbilisi, 0177 Republic of Georgia

**Keywords:** Epilepsy, Medicinal herbs, Biomarkers, Antiseizure medications, Ion channels, mTOR signaling, Neuroprotection

## Abstract

Epilepsy is a neurological disorder characterized by spontaneous and recurring seizures. It poses significant therapeutic challenges due to diverse etiology, pathobiology, and pharmacotherapy-resistant variants. The anticonvulsive effects of herbal leads with biocompatibility and toxicity considerations have attracted much interest, inspiring mechanistic analysis with the view of their use for engagement of new targets and combination with antiseizure pharmacotherapies. This article presents a comprehensive overview of the key molecular players and putative action mechanisms of the most common antiepileptic herbals demonstrated in tissue culture and preclinical models. From the review of the literature, it emerges that their effects are mediated via five distinct mechanisms: (1) reduction of membrane excitability through inhibition of cation channels, (2) improvement of mitochondrial functions with antioxidant effects, (3) enhancement in synaptic transmission mediated by GABA_A_ receptors, (4) improvement of immune response with anti-inflammatory action, and (5) suppression of protein synthesis and metabolism. While some of the primary targets and action mechanisms of herbal anticonvulsants (1, 3) are shared with antiseizure pharmacotherapies, herbal leads also engage with distinct mechanisms (2, 4, and 5), suggesting new drug targets and opportunities for their integration with antiseizure medications. Addressing outstanding questions through research and in silico modeling should facilitate the future use of herbals as auxiliary therapy in epilepsy and guide the development of treatment of pharmacoresistant seizures through rigorous trials and regulatory approval.

## Introduction

Epilepsy is one of the most common and potentially fatal neurological conditions affecting ~ 70 million individuals worldwide. According to the World Health Organization, the average incidence of epilepsy in industrialized countries is ~ 50 per 100,000 population, with nearly double of that in developing countries [1, 2]. Despite remarkable progress in research and development of effective pharmacotherapies, in 20–30% of cases, antiepileptic drugs fail to control clinical seizures [3–5]. Affecting one in four of the general population of patients, pharmacoresistant epilepsy is associated with high risks of injuries related to seizures and major psychosocial trauma, occasionally leading to disability and premature death [6, 7]. Moreover, the majority of antiseizure medications (ASM) have multiple adverse neurological effects, including drowsiness, headaches, dizziness, agitation, blurred vision, and uncontrollable shaking, calling for new medicines that are tolerated better and have minimal side effects [8, 9]. Notably, some of the frontline antiseizure pharmacotherapies may also cause endocrinal disturbances, with effects particularly detrimental in children, which may have lasting unfavorable consequences [10, 11]. Analysis of the impact of long-term use of ASM on thyroid functions of children, for instance, revealed a reduction in thyroxine (T_4_) and triiodothyronine (T_3_) levels in serum by some, implying the contribution of thyroid dysfunctions [12]. With described untoward effects and lack of response to antiseizure medications in 20-30% subjects, there is a pressing need for mechanistic research to pursue new and affordable therapeutics, aiming not only at better management of seizures but also at more natural and cost-effective interventions with fewer side effects. To this end, anticonvulsant herbals used over millennia have generated renewed interest [13, 14]. Utilized mainly as stand-alone treatment, herbals with anticonvulsant properties have displayed several advantages over pharmacotherapies, demonstrating superior biocompatibility, less toxicity, and milder neurological side effects [13, 15, 16]. As natural products, anticonvulsant herbals are also more affordable and are increasingly used in developing countries [17, 18]. Remarkably, in addition to suppressing epileptic seizures through a direct stabilizing effect on neuronal excitability by acting on ion channels and neurotransmitter receptors, herbal anticonvulsants have also been reported to alleviate neuroinflammation and immune response in the brain, inhibit autophagy, and improve the homeostatic processes [19, 20]. These additional outcomes have been suggested to contribute to the secondary antiseizure actions of herbal anticonvulsants, with increasing recognition of their relevance to therapy of pharmaco-resistant variants of epilepsy [20–22].

Although, in present days, the practice of antiseizure herbals for the therapy of epilepsy is primarily based on established traditions and anecdotal efficacy reports, some have passed rigorous scientific trials and have been licensed by the US Food and Drug Administration as anti-epilepsy leads [23, 24]. As it emerges from present discussions of in vitro and in vivo preclinical studies, the antiepileptic effects of herbals are mediated via five distinct mechanisms: (1) attenuation of the neuronal excitability through modulation of ion channel activity, (2) enhancement of mitochondrial functions with antioxidant effects, (3) allosteric enhancement of GABA_A_ receptor mediated currents, (4) suppression of inflammatory response, and (5) modulation of protein synthesis and metabolism. While some of these effects (1, 3) are shared with established antiseizure drugs, others (2, 4, and 5) are specific to herbal anticonvulsants, suggestive of novel targets for pharmacotherapies as well as opportunities of their combination with ASM for additive effects.

This article presents a comprehensive overview of the putative action mechanisms and targets of the most widely used antiepileptic herbals in tissue culture and animal models. The emerging data not only advocate the possible uses of herbal leads as adjuvants to antiseizure drugs but also highlight their potential as a guide for drug discovery efforts towards new leads and targets relevant to refractory forms of epilepsy.

## Methods

All authors conducted a literature search using scientific databases such as PubMed and ScienceDirect. Where necessary, Google Scholar, Academia, and ResearchGate have been used as additional sources of information. Keywords for search were antiepileptic herbals; antiseizure plant medicine; herbal anticonvulsant; traditional medicine for epilepsy; animal models of epilepsy; pharmaco-resistant epilepsy and herbal therapies; epilepsy biomarkers; ion channels and herbal medicine; mTOR signalling and epilepsy; herbal neuroprotection. The reference list of articles was scanned to identify further information relevant to the current analysis. A summary of all references was drafted, followed by thematic grouping and manuscript writing. Figures are prepared using Adobe Illustrator Artwork 16.0 of the Adobe Creative Suit version 6.0. The table has been generated using Microsoft Word. EndNote X8.2 was used for citations, with references formatted per Journal Guidelines.

### Anticonvulsant herbals reducing neuronal excitability

**Fig. 1:**
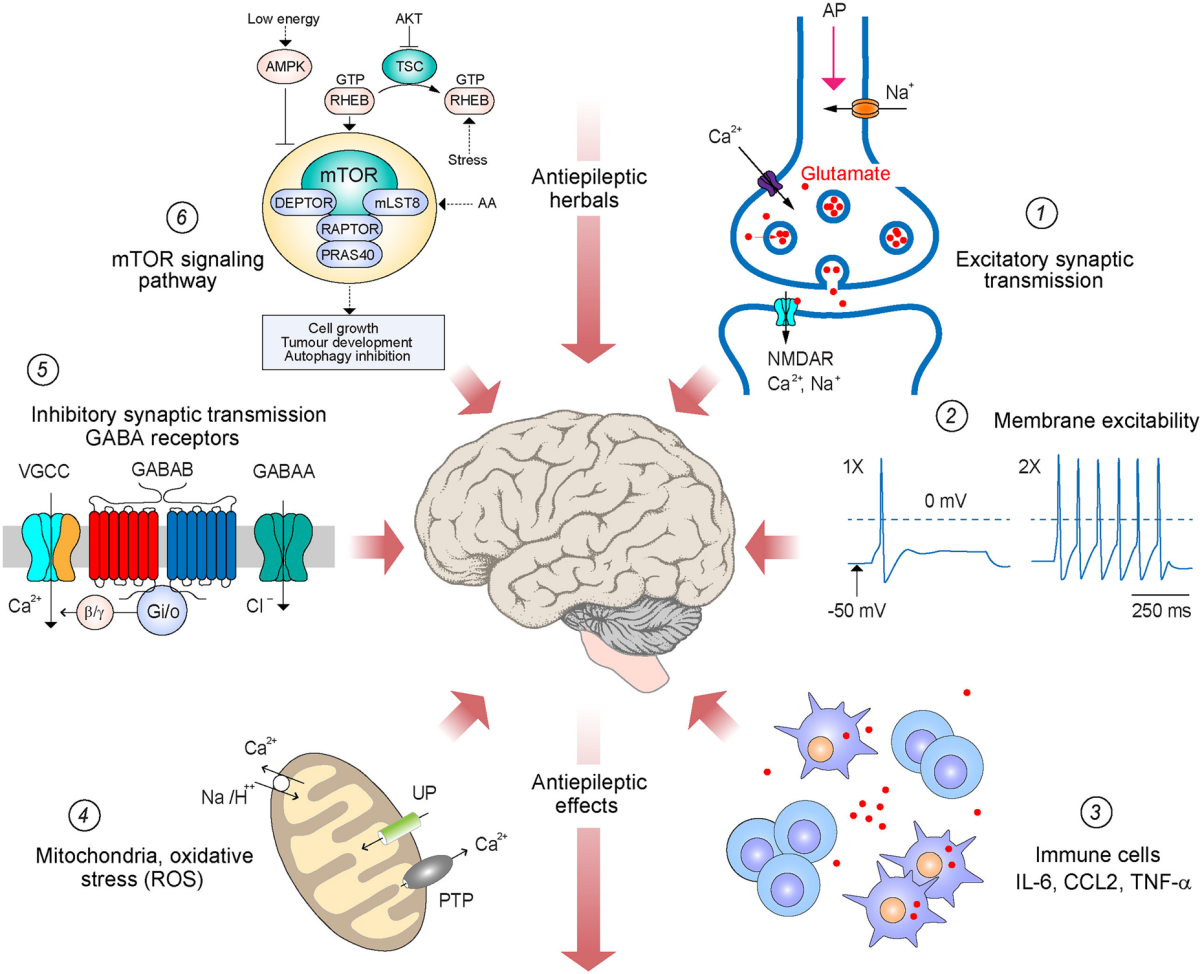
Schematic representation of major targets and putative mechanisms implicated in antiepileptic effects of herbals. (1) Synaptic transmission at glutamatergic connections, post-synaptic NMDA receptor, presynaptic voltage-gated Ca^2+^, and voltage-gated Na^+^ channels; (2) membrane excitability and action potential firing through modulation of ion channel activity; (3) neuroimmune and inflammatory response in the brain; (4) enhancement of mitochondrial functions and restoring ROS signaling; (5) modulation of inhibitory neurotransmission by altering GABAergic drive; and (6) downregulation of mTOR signaling. TSC: tuberous sclerosis complex; GTP: guanosine 3-phosphate; AA: amino acids; AMPK: AMP-activated protein kinase; AKT: protein kinase B; RHEB: Ras homolog enriched in brain; DEPTOR: DEP domain-containing mTOR-interacting protein; RAPTOR: regulatory-associated protein of mTOR; PRAS40: proline-rich Akt substrate of 40 kDa

**Fig. 2:**
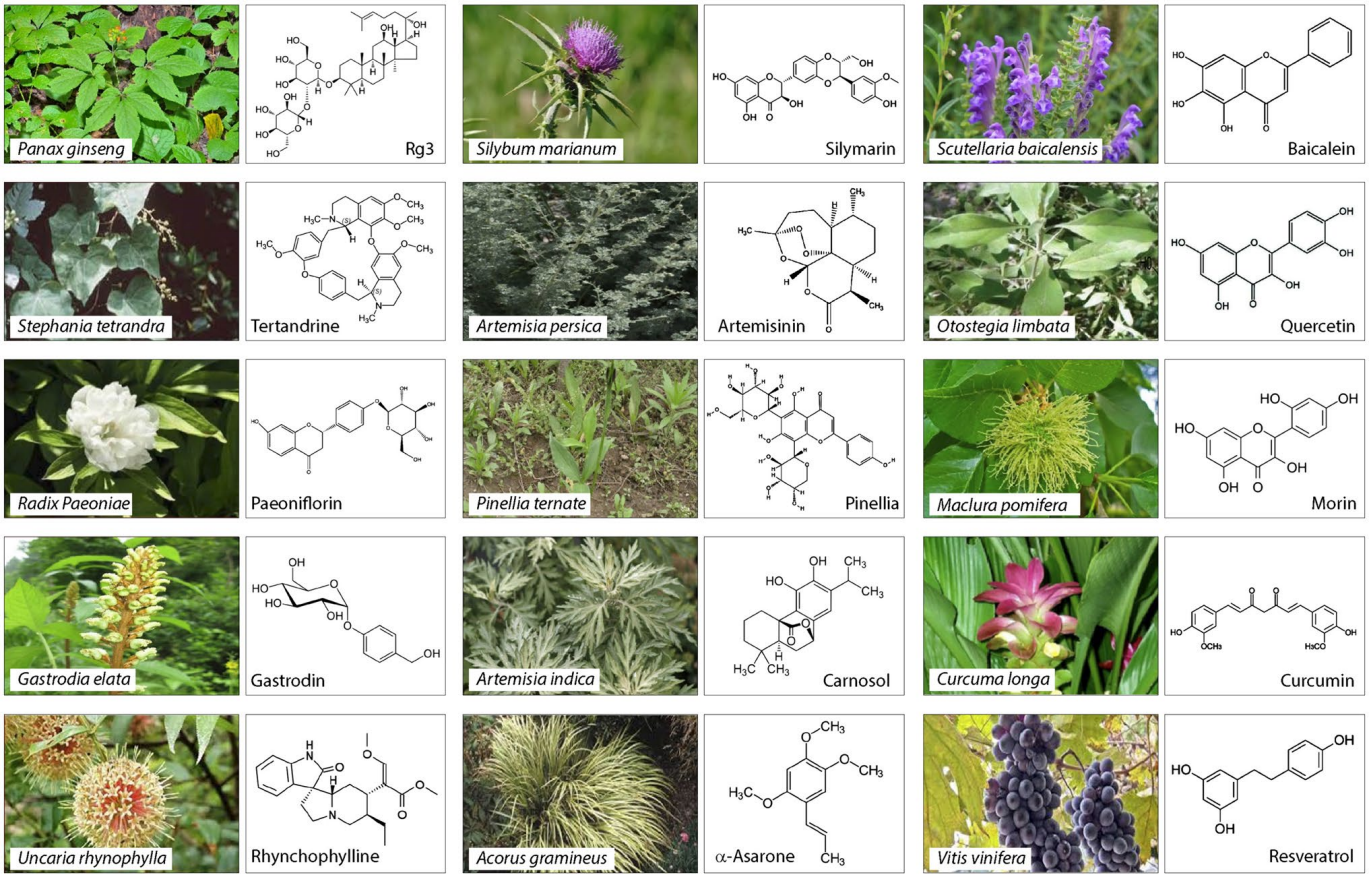
Illustration of selected medicinal herbals with anticonvulsant effects and chemical structure of the active lead. Individual panels are taken from Britannica plants: https://www.britannica.com/plant/ginseng

Abnormal activity of ion channels and receptors accounts for a significant portion of epilepsy cases, with membrane hyperexcitability linked to the initiation and propagation of seizures [25–27]. Most antiepileptic effects of medicinal plants acting on ion channels and receptors are attributed to lowering the excitability of glutamatergic neurons, with attenuation of excitatory transmission [28, 29] (Fig. 1). Several studies demonstrated the antiseizure effects of ginsenoside Rg3 enriched in *Panax ginseng*, capable of restoring Ca^2+^ homeostasis by inhibiting the *N*-methyl-*D*-aspartate (NMDA) glutamatergic receptor [30, 31] (Fig. 2). In epilepsy models of cultured hippocampal neurons, Rg3 inhibits intracellular Ca^2+^ oscillations and lowers its concentration by suppressing Ca^2+^ influx mediated by NMDA receptor [31]. These effects suggest that the anticonvulsive activity of Rg3 could also counter cytotoxicity related to high intracellular Ca^2+^ caused by NMDA receptor hyperactivity in status epilepticus (SE) or during spontaneous recurrent epileptiform discharges (SREDs) [31]. Inhibition of Ca^2+^ influx with attenuation of the activity of glutamatergic neurons also underlies the anticonvulsant effects of *Stephania tetrandra* [32]. Unlike Rg3 acting upon the NMDA receptor, the active lead of *S. tetrandra*, tetrandrine, blocks voltage-gated calcium channels (VGCC), as reported in several excitable cells, including rat neurohypophysial nerve terminals [32] (Fig. 2). Notably, tetrandrine can improve the antiepileptic effects of antiseizure pharmacotherapies such as phenytoin and valproate on milder seizures in refractory epilepsy models in rats induced by doxorubicin. This effect appears to be partly mediated via lowering the expression of multidrug-resistant P-glycoprotein in tested cortical and hippocampal neurons [33]. Inhibition of Ca^2+^ influx is also thought to contribute to tempering the hyperexcitability associated with febrile seizures (FS) by *Radix paeoniae*, with paeoniflorin as the active ingredient [34] (Fig. 2). In the hyperthermia-induced FS model of immature rats, paeoniflorin inhibited the glutamate-induced elevation of intracellular Ca^2+^ with membrane depolarization and cytotoxicity associated with the hyperactivity of NMDA receptors [34].

Voltage-gated sodium channels (VGSC) are also an important drug target for managing epilepsy [35, 36]. Hyperactivity of VGSC is essential in the generation and propagation of seizures. Therefore, suppressing Na^+^ influx in neurons by herbals is anticipated to have antiepileptic effects [13, 37]. Gastrodin, a phenolic glucoside from *Gastrodia elata* has been shown to exhibit antiepileptic activity by attenuating Na^+^ currents, reducing the severity and duration of SE in the pilocarpine rat model of temporal lobe epilepsy (TLE) [38] (Figs. 1 and 2). This effect protected neurons of the layer III medial entorhinal cortex from degeneration in SE and lowered the damage to neurons of the layer II medial entorhinal cortex (mEC). In addition, gastrodin could attenuate the inflammatory responses to pentylenetetrazol (PTZ)-induced seizures in mice [39]. Similar effects but through inhibition of specific subtype of Na^+^ channels have been reported for rhynchophylline (RIN), an alkaloid derived from *Uncaria rhynchophylla*, which showed a potent anticonvulsant effect in a pilocarpine-induced SE rat model of TLE [40]. The tempering effect of RIN on neuronal hyperexcitability was mediated via inhibition of persistent sodium current (*I*_NaP_) and NMDA receptor. Furthermore, RIN has shown protective effects on medial entorhinal cortex layer III neurons and inhibited spontaneous epileptiform discharge of mEC layer II neurons in SE rats [40]. Finally, antiepileptic effects via dual NMDA receptor and *I*_NaP_ targeting have been described for saikosaponin A (SSA) isolated from *Radix bupleuri* [41]. Such binary action was especially effective in terminating spontaneous recurrent epileptiform discharges in the hippocampal neuron culture (HNC) model of acquired epilepsy and continuous epileptiform high-frequency bursts of SE [42] (Table 1).

**Table 1:** Summary of reviewed medicinal plants demonstrating anticonvulsant activity in preclinical models, with active leads, molecular targets, action mechanisms and corresponding references

**Medicinal plants**	**Compounds**	**Targets**	**Action mechanisms**	**Preclinical models**	**References**
*Panax ginseng*	Rg3	NMDAR	Inhibition/NMDAR	Neuronal culture	[27]
*Stephania tetrandra*	Tetrandrine	VGCC	Blockade/Ca^2+^	Rat (nerve terminals) Doxorubicin model	[28, 29]
*Radix paeoniae*	Paeoniflorin (PF)	NMDAR	Inhibition/NMDAR	Rat (hyperthermia febrile seizures model)	[30]
*Gastrodia elata*	Gastrodin (GAS)	VGSC; free radicals	Blockade/Na^+^ Antioxidant	Rat (pilocarpine TLE model; KA model)	[34, 43]
*Uncaria rhynchophylla*	Rhynchophylline (RIN)	NMDAR	Inhibition/Na^+^	Rat (pilocarpine SE model)	[36]
*Radix bupleuri*	Saikosaponin A (SSA)	NMDAR; mTOR	Inhibition/Na^+^; mTOR	Neuronal culture Rat (PTZ model)	[38, 44]
*Artemisia persica*	Artemisinin	Free radicals; Inflammation	Anti-oxidation, IL-1β, TNF-α inhibition	Mouse (PTZ model)	[45]
*Silybum marianum*	Silymarin	Free radicals	Anti-oxidation	Mouse (PTZ model)	[46]
*Pinellia ternate*	Pinellia	GABAAR	Activation	Rat (pilocarpine model)	[47]
*Artemisia indica*	Carnosol, ursolic & oleanolic acid	GABAAR	Activation	Mouse (PTZ model)	[48]
*Acorus gramineus*	α-Asarone	GABAAR NMDAR, VGSC	Activation GABAAR Blockade/Na^+^	Mouse (PTZ model)	[49]
*Scutellaria, baicalensis*	Baicalein	IGF-1 receptor Inflammation	Attenuation of IL-1β, IL-6, and TNF-α expression	Rat (pilocarpine model)	[50]
*Ruta graveolens*	Quercetin, Rutin	TLR4/NF-kB Inflammation	IL-1β, IL-6, TNF-α inhibition, HMGB1 expression	Rat (KA model)	[51]
*Citrus limon*	Hesperidin	Inflammation	BDNF and IL-10 inhibition	Zebrafish larvae (PTZ model)	[52]
*Otostegia limbata*	Quercetin, Rutin	Inflammation	TNF-α and p-NF-Kβ inhibition	Mouse (PTZ model)	[53]
*Maclura pomifera*	Morin	mTOR	Inhibition	Mouse (KA model)	[54]
*Curcuma longa*	Curcumin	mTOR	Inhibition	Neuronal culture	[55]
*Vitis vinifera*	Resveratrol	AMPK/mTOR	Inhibition	Rat (pilocarpine model)	[56]

### Anticonvulsant herbals with antioxidant effects

Reactive oxygen species (ROS) are the leading cause of oxidative stress, which can contribute to the hyperexcitability of glutamatergic neurons and epileptogenesis [57, 58]. ROS play a critical role in the molecular signaling of neurons, regulating neuronal activity, membrane excitability, and synaptic plasticity mechanisms (Fig. 1). When in excess, ROS become highly toxic, interfering with various physiological processes and functions [55, 59]. In epilepsy, oxidative stress with increased ROS is explored as one of the early biomarkers contributing to the generation of seizures and cytotoxicity. Substantial data suggests that oxidative stress might be especially prominent in refractory epilepsy, with specific mechanisms remaining elusive [60, 61]. Therefore, restoring the oxidative balance using pharmacotherapies and herbal medicines might tamper epileptic seizures and avert neuronal damage with cytotoxicity [45, 46].

The antioxidant effects of *Artemisia* extracts with the prevention of oxidative stress and neuroprotection via modulation of the endogenous antioxidants have been shown by several groups [62, 63] (Fig. 2, Table 1). Analysis of the effects of hydroalcoholic compounds of *A. persica* on PTZ-induced seizures and memory impairments in experimental mice have shown amelioration of convulsions and memory improvements [43]. In the same model, the extracts of *Artemisia* increased the antioxidant capacity of the serum and brain tissue. These favorable outcomes correlate with the reduced frequency of spinning and jumping of epileptic mice and the attenuation of their tonic seizures. Interestingly, the anticonvulsant effects of *A. persica* extracts can be enhanced by diazepam, while flumazenil has the opposite effect. Histological examination of brain tissue showed reduced IL-1β and TNF-α expression by *A. persica* in the brain of the PTZ-treated mouse model [43]. In vitro antioxidant effects of the hydroalcoholic *A. persica* extracts was also verified by using tests for oxidative activity, which demonstrated enhancement in 2,2-diphenyl-1-picrylhydrazyl (DPPH) free radicals scavenging, stabilization of 2,2′-azino-bis 3-ethylbenzothiazoline-6-sulfonic acid (ABTS) activity and hydroxyl free radicals scavenging, as well as restoration of Fe^3+^ level with higher ferric ion reducing activity [43].

*Silybum marianum* is another anticonvulsive herbal with antioxidant and antiseizure effects, lowering the frequency and duration of convulsions in PTZ experimental mice and reducing PTZ-induced lethality [64] (Fig. 2). This effect was mediated partly via attenuation of the oxidative stress in the brain with the increase in superoxide dismutase and catalase activity and a reduced level of lipid peroxidation [64]. Finally, antioxidant effects have also been implicated in the ameliorative influence of *Gastrodia elata* on several neurological conditions. As a traditional Chinese medical herb, *G. elata* with active ingredient gastrodin demonstrated promising therapeutic outcomes in preclinical models of epilepsy, Alzheimer’s disease, and Parkinson’s disease [65, 66]. The anticonvulsive effects of *G. eleta* were reported in the kainic acid (KA) rat model, potentially related to its free radical scavenging [67] (Fig. 2). The oral administration of *G. eleta* significantly and dose-dependently reduced the level of lipid peroxides in the rat brain, leading to fewer incidents of wet dog shakes, paw tremors, and facial myoclonia [67].

### Anticonvulsant herbals targeting γ-aminobutyric acid (GABA) receptors

The disbalance of excitatory/inhibitory (E/I) synaptic activity in cortical and hippocampal glutamatergic neurons plays a crucial role in the pathobiology of several neurological and neurodegenerative conditions [68–71]. In epilepsy, this change leads to the disinhibition of neuronal networks, resulting in paroxysmal hyperactivity, profuse glutamate release and associated cytotoxicity [4, 39]. GABA is the most prevalent inhibitory neurotransmitter in the central nervous system, stabilizing neuronal activity in the brain and spinal cord, with a decrease in GABAergic synaptic drive leading to epileptogenesis [47, 72]. As a transmitter, GABA acts via three groups of specific receptors: GABA_A_, GABA_B_, and GABA_C_ [48, 49]. Upon binding to GABA, GABA_A_ receptors mediate Cl^−^ influx across the membrane, leading to neuronal hyperpolarization and reduction in excitability, preventing spontaneous and induced seizures in animal studies and epilepsy patients [73] (Fig. 1).

Several medicinal plants produce and accumulate large amounts of metabolites known as flavonoids, which share structural similarities with positive modulators of GABA_A_ receptors - benzodiazepines [74]. Like benzodiazepines, these flavonoids are potent anticonvulsants, acting primarily as GABA_A_ receptor enhancers, potentiating inhibitory synaptic currents [75]. One of the most utilized antiseizure herbs, *Pinellia ternate*, contains high concentrations of *Pinellia* total alkaloids (PTA), which enhances GABA_A_ currents, preventing pilocarpine-induced convulsions in a rat model [76] (Fig. 2, Table 1). As a result, the incidence and intensity of seizures are significantly reduced compared to pilocarpine-treated rats without PTA. Notably, prolonged exposure to PTA led to higher expression of GABA_A_ receptors and suppressed the levels of GABA transporter-1 (GAT-1). The mRNA analysis of GABA_A_ receptor showed upregulation of low-expressed mRNA of GABA_A_R α5 subunit, δ subunit and γ2 subunit along with enhancement of glutamate decarboxylase 65 (GAD65), while GAT-1, GABA transaminase (GABA-T) mRNA levels showed opposite trend [76].

An earlier study isolated and characterized three active ingredients, carnosol, ursolic acid, and oleanolic acid, from another antiepileptic plan, *Artemisia indica*, acting on GABA_A_ receptors with anticonvulsant effects [77] (Fig. 1). These substances have enhanced the response of GABA_A_ receptors to GABA in a concentration-dependent manner, operating as a positive modulator of α1β2γ2L containing GABA_A_ receptors at the benzodiazepine binding site [77]. Analysis of the antiepileptic activity of carnosol, ursolic acid, and oleanolic acid on PTZ-induced convulsions in mice showed that all three have potent antiseizure effects [77]. Finally, α-asarone is another widely recognized antiepileptic herbal extract targeting GABA_A_ receptors. Isolated from *Acorus gramineu*s, α-asarone has been successfully used in clinical practice to treat various epileptic seizures [78]. Detailed neurobiological analysis showed that α-asarone combines potent antiseizure activity with neuroprotective, antipyretic, and analgesic effects, offering therapeutic potential against epilepsy, depression and anxiety [79]. Its antiepileptic effects are mediated via multitarget actions, including the enhancement of GABA_A_ receptor activity, inhibition of glutamatergic drive mediated via NMDA receptors, and block of VGSC [80]. In PTZ and kainite mouse models, α-asarone strongly inhibits the excitability of hippocampal neurons, prolonging the latency of clonic and tonic seizures and reducing animal mortality [78] (Table 1).

### Anticonvulsant herbals targeting neuroinflammation

Increasing preclinical and clinical data support the role of neuroinflammation in epileptogenesis [21, 81]. As part of the inflammatory response, glial cells release cytokines and proinflammatory factors, exacerbating the disease process and promoting epileptic seizures [50, 82]. Lowering the level of cytokines and inflammatory factors is, therefore, expected to restore the physiological activity of neuronal networks and suppress epileptogenesis (Fig. 1).

Several medicinal plants rich in flavonoids were shown to counter inflammation by lowering proinflammatory factors and cytokines [51, 83] (Fig. 2, Table 1). Baicalein, a flavone from *Scutellaria baicalensis* and *Scutellaria lateriflora*, has ameliorative effects on seizure propensity and cognitive deficits in epilepsy models [52, 53]. In posttraumatic epilepsy, which is acquired epilepsy secondary to traumatic brain injury and has a significant inflammatory component, baicalein exerts neuroprotective and antiepileptic effects [53]. It also significantly reduced the number and scores of seizures and average seizure duration in an iron chloride (FeCl_3_)-induced posttraumatic epilepsy (PTE) mouse model. The neuroprotective effects of baicalein were also reported in ferric ammonium citrate (FAC)-induced HT22 hippocampal injury model, where baicalein reduced ferroptotic indices in vitro and suppressed the expression of 12/15-lipoxygenase (12/15-LOX) in an iron-induced HT22 cell damage model. These findings support baicalein’s neuroprotective and anticonvulsive effects on PTE [53]. Likewise, in pilocarpine-induced epileptic rats, oral administration of baicalein dose-dependently decreased epilepsy symptoms, which was associated with inhibition of microglial proliferation, reduction in inflammatory markers, such as IL-1β, IL-6, and TNF-α in the brain [84].

Another flavonoid, quercetin, which is enriched in *Allium cepa* [85, 86] and several common fruits and vegetables, has shown potent anti-inflammatory action by inhibiting the TLR4/NF-kB signaling in animal models of epilepsy [85, 86]. In the same vein, in KA-injected rats, rutin from *Ruta graveolens*, attenuated the release of inflammatory molecules like IL-1β, IL-6, TNF-α, and high-mobility group box 1 (HMGB1) protein and suppressed the IL-1R1 and toll-like receptor 4 (TLR4) expression, preventing seizures [87]. A similar analysis of the effects of hesperidin, a flavonoid found in citrus fruits, showed longer latency with reduced intensity of PTZ-induced seizures in zebrafish larvae [88]. The anticonvulsant effects of HMGB1 were associated with changes in the expression of brain-derived neurotrophic factor (BDNF) and IL-10. Of note, hesperidin has a strong affinity for several receptors, including IL-10, as suggested by *in-silico* studies [88].

A recent analysis of the effects of methanolic extracts of *Otostegia limbata* on the expression of TNF-α and phosphorylated transcription factor nuclear factor kappa B (p-NF-κB) in the cortex and hippocampus of the PTZ-induced convulsion model showed significant anti-inflammatory effects associated with shorter duration and increased latency of seizures [56] (Fig. 2, Table 1). TNF-α and p-NF-κB expressions were strongly reduced compared to the vehicle-untreated control group. The authors suggested that the effects of *O. limbata* are attributed to high quantities of phenols and flavonoids, which demonstrated anticonvulsant effects in preclinical models of epilepsy.

### Anticonvulsant herbals targeting mTOR signaling

The mammalian target of the rapamycin (mTOR) signaling pathway, which controls cell growth, differentiation, proliferation, and metabolism [46, 54], is another putative target of antiepileptic herbal leads (Fig. 1). Pathological changes causing hyperactivity of mTOR signaling are known to lead to neuronal dysplasia with neurodevelopmental disorders, including autism and epilepsy [44, 89–91]. Other etiologies of epilepsy, such as brain injury and neonatal hypoxia–ischemia, have also been linked to hyperactivity of the mTOR pathway [92].

Several substances extracted from herbs have been reported to inhibit mTOR signaling [20, 93]. Morin, a phytochemical derived from a variety of plants, including *Maclura pomifera*, *M. tinctoria*, and *Psidium guajava*, has been shown to lower the vulnerability of preclinical models to epileptic seizures, an effect associated with reduction in expression of apoptotic molecules and inflammatory cytokines (Fig. 2, Table 1). In KA-induced seizure studies, morin caused significant inhibition of mTOR complex 1 (mTORC1) and decreased the propensity of mice to develop generalized seizures [94]. These effects were associated with reduced granule cell dispersion and suppression of mossy fiber sprouting in the hippocampus seven days after KA treatment [94]. Inhibition of mTOR signaling has also been implicated in the antiepileptic effects of SSAs in PTZ-induced rat models of epilepsy [95]. These oleanane-based glycosides, abundantly present in *Radix bupleuri* and several other medicinal plants, significantly reduce the severity and duration of seizures in PTZ models and increase the seizure latency [95]. The anticonvulsive effects of glycosides are associated with downregulation in p-mTOR, p-70S6K, L-1β, and TNF-α expression in hippocampal neurons of PTZ rat models [95]. Inhibition of mTOR activity and associated downregulation of the mitogen-activated kinase signaling also emerges to be involved in antiseizure effects of curcumin, an active phytochemical constituent of the medicinal herb *Curcuma longa* [46]. Quantitative real-time PCR analysis revealed that curcumin, but not rapamycin, reduced inflammatory markers IL-6 and COX-2 levels in cultured astrocytes challenged with IL-1β. In SH-SY5Y cells, curcumin reduced ROS levels, suggesting combined antioxidant and anti-inflammatory effects, with secondary antiepileptic outcomes. In the SE rat model, however, treatment with rapamycin or curcumin did not lower the inflammatory and oxidative stress markers up to one week after SE [46].

Resveratrol is another natural medicinal plant extract polyphenol with antiepileptic effects, curtailing the recurrence of seizures and SE in preclinical models [96, 97]. Its antiseizure effects seem to involve inhibiting NF-kB and suppressing proinflammatory cytokine synthesis, which can be influenced by upstream mTOR signaling [54, 98]. In pilocarpine-induced seizures in a rat model, resveratrol isolated from *Vitis vinifera* lowered COX-2, IL-1β and inducible NO synthase levels in the hippocampus 3 h after SE [93] (Fig. 2). Pretreatment of rats with resveratrol before pilocarpine-induced seizures also increased the expression of AMP-activated protein kinase, which was associated with a reduction in phosphor-mTOR and target phospho-S6 (pS6) protein, implying dual anti-inflammatory and antiepileptic effects, which involve AMPK/mTOR signaling pathway.

## Summary and conclusions

Nearly half of the pharmaceutical products used in medicinal practice today draw from traditional medicine and natural remedies, with many delivering life-saving solutions. The isolation of artemisinin from sweet wormwood by Tu and co-workers [99] to treat quinidine-resistant malaria presents an iconic example of a cure by natural remedies used in traditional medicine, saving millions of lives. The impact of this breakthrough has been recognized by the Nobel Prize for Physiology and Medicine of 2015 [100, 101], with the World Health Organization recommending artemisinin as the first- and second-line malaria treatment.

Unlike quinidine-resistant malaria treated nowadays by traditional medicine, many devastating conditions, which also include refractory epilepsy, are resistant to pharmacotherapies and have no natural solution. Research on herbal anticonvulsants offering relief from epileptic seizures, therefore, is viewed as one of the promising avenues towards enabling effective intervention. In this analysis, we considered the emerging data from in vitro and preclinical studies of herbal leads in animal models showing promise for managing epileptic seizures. In addition to engaging with ion channels and neurotransmitter receptors, the evolving data suggests herbal-induced improvements in mitochondrial functions with antioxidant effects, attenuation of neuroinflammatory response and immune processes, and alterations in protein synthesis and metabolism as mechanisms for beneficial outcomes (Fig. 1). Given the limited efficacy of frontline antiseizure pharmacotherapies acting upon neurotransmission and excitability of neurons in refractory epilepsy, engagement with additional targets is well warranted and likely to yield beneficial effects [4, 5, 61, 92, 102]. Discussed herein studies in neuronal cultures and preclinical models suggest that ameliorative outcomes of herbals may result from their use as monotherapy and in combination with conventional antiepileptic pharmacotherapies. Both approaches necessitate rigorous dose–response, efficacy, and safety considerations, along with the review of the specifics related to individual variability and potential co-morbid conditions. In tandem with studies of clinical biomarkers and in silico analysis, these developments should facilitate understanding the neurobiological mechanisms of refractory epilepsy to guide the discovery of antiseizure leads with better efficacy, safety, and cost outcomes.

In conclusion, the future of herbal treatments of epilepsy might hold significant mechanistic and translational insights towards developing innovative primary therapies and adjuvants for integration with antiseizure medications. Guided by herbal leads, the drug discovery process will also likely facilitate the detection of synthetic analogues and modified leads to meet therapeutic needs through well-designed clinical trials and rigorous regulatory approval.

## Data Availability

This is not applicable. If accepted for publication, the material will be made freely available to the public.

## Abbreviations

ASM: Antiseizure medications
NMDA: *N*-Methyl-*D*-aspartate
SE: Status epilepticus
SREDs: Spontaneous recurrent epileptiform discharges
VGCC: Voltage-gated calcium channels
FS: Febrile seizures
VGSC: Voltage-gated sodium channels
TLE: Temporal lobe epilepsy
mEC: Medial entorhinal cortex
PTZ: Pentylenetetrazol
RIN: Rhynchophylline
*I*_NaP_: Persistent sodium current
SSA: Saikosaponin A
HNC: Hippocampal neuron culture
ROS: Reactive oxygen species
TNF: Tumor necrosis factor
IL: Interleukin
DPPH: 2,2-Diphenyl-1-picrylhydrazyl
ANTS: 2,2′-Azino-bis 3-ethylbenzothiazoline-6-sulfonic acid
KA: Kainic acid
E/I: Excitatory–inhibitory ratio
GABA: γ-Ammino butyric acid
PTA: Pinellia total alkaloids
GAT-1: GABA transporter-1
GAD65: Glutamate decarboxylase 65
GABA-T: GABA transaminase
PTE: Posttraumatic epilepsy
FAC: Ferric ammonium citrate
12/15-LOX: 12/15-Lipoxygenase
HMGB1: High-mobility group box 1
BDNF: Brain-derived neurotrophic factor
p-NF-κB: Phosphorylated transcription factor nuclear factor kappa B
mTOR: Mammalian target of the rapamycin
PCR: Polymeric chain reaction
COX-2: Cyclooxygenase 2
NO: Nitric oxide
